# Quality comparison between two different types of platelet-rich plasma for knee osteoarthritis

**DOI:** 10.1051/rmr/200002

**Published:** 2020-12-03

**Authors:** Takanori Wakayama, Yoshitomo Saita, Yohei Kobayashi, Hirofumi Nishio, Sayuri Uchino, Shin Fukusato, Hiroshi Ikeda, Kazuo Kaneko

**Affiliations:** 1 Department of Orthopaedics, Juntendo University, Faculty of Medicine, 2-1-1 Hongo Bunkyo-ku Tokyo 113-8421 Japan; 2 Department of Physical Therapy, Juntendo University, Faculty of Health Science, 2-1-1 Hongo Bunkyo-ku Tokyo 113-8421 Japan

**Keywords:** Autologous protein solution, platelet-rich plasma, leukocyte, platelet, anti-inflammatory cytokine, growth factor

## Abstract

*Introduction:* Knee osteoarthritis (KOA), the most common form of osteoarthritis (OA) is a considerable health concern worldwide. Platelet-rich plasma (PRP) is a common therapeutic option for KOA. Different types of PRPs have varying efficacies. However, a comparative analysis of the qualities of these PRPs is lacking. *Methods:* Two types of PRPs, including autologous protein solution (APS), and leukocyte-poor PRP (LP-PRP) along with whole blood (WB) and platelet-poor plasma (PPP) were characterized for platelet content, leukocyte content, and composition in 10 healthy volunteers (HV) (the controlled laboratory study) and 16 KOA patients (a retrospective observational study). Additionally, the levels of the platelet-derived growth factor (PDGF)-BB, and different cytokines were estimated in HV. *Results:* In HV, the concentrations of platelets and leukocytes, levels of different cytokines, including interleukin 1 receptor antagonist (IL-1Ra), soluble TNF receptor type II (sTNF-RII), and IL-1β, and the ratio of IL-1Ra/IL-1β were significantly higher in APS, whereas the PDGF-BB was higher in LP-PRP than APS. In KOA patients, a higher concentration of platelets was observed in LP-PRP, and a higher concentration of leukocytes was observed in APS than LP-PRP. Following the PAW classification system, LP-PRP was classified as P2-B type in HV (51.3 × 10^4^/μl) and KOA (53.4 × 10^4^/μl), whereas APS was classified as P3-A type in HV (110.1 × 10^4^/μl) and P2-A type in KOA (29.0 × 10^4^/μl). In a retrospective observational study, the KOA patients who underwent APS injection had a higher incidence of arthralgia, and this arthralgia lasted for a longer time than LP-PRP injection in the same individual. *Discussion:* The quality of the two PRPs differed distinctively depending on their preparation methods, which might affect their clinical efficacies and adverse events. Therefore, the characterization of these parameters should be prioritized while choosing PRP.

## 1 Introduction

Osteoarthritis (OA) is the most common form of arthritis, which affects the knee joint more often than the other joints in older people. The knee osteoarthritis (KOA) has become a major health concern because of the difficulties for its treatment. Therefore, it is imperative to evaluate safe and effective treatment methods, and orthobiologics, representing a relatively new area of cell-based therapy, have captured the interest of orthopedic surgeons.

Orthobiologics are natural substances, such as cells, blood components, and growth factors, which are used to promote the healing of soft and hard tissues such as muscle, cartilage, ligament, tendon, and bone tissues [1,2]. Among them, platelet-rich plasma (PRP)—an autologous platelet concentrate containing diverse growth factors such as platelet-derived growth factor (PDGF), transforming growth factor-beta (TGF-β), and vascular endothelial growth factor (VEGF) and other cytokines and enzymes exerting not only anabolic but also catabolic effects [3–5]—has several advantages over other orthobiologics. It is a minimally-invasive, safe, and simple therapy that is associated with fewer side effects [6]. Several types of PRP preparation kits are commercially available, which differ with respect to the methods of extraction, platelet, and other blood component concentrations. Therefore, it is very important to analyze the quality of PRPs for their use in orthobiological treatments. Several randomized clinical trials, each using different PRPs, have revealed the effectiveness of PRP therapy for the treatment of KOA; however, the level of evidence was not high [7–10].

Several studies have tried to characterize and classify PRPs. DeLong et al. [11] proposed the PAW classification system based on platelet concentration (P), activation status (A), and white blood cell concentration (W); whereas, Dohan Ehrenfest et al. [12] classified PRPs into three categories, leukocyte-rich (LR)-PRP, leukocyte-poor (LP)-PRP, and pure-PRP, based on the concentration of white blood cells.

The activities of PRPs have been shown to be dependent on cell compositions, and especially leukocyte concentrations [13]. Moreover, LP-PRP has been hypothesized to be more suitable for intra-articular injection and is most frequently used than LR-PRP in the treatment of KOA [14]. Additionally, autologous protein solution (APS; dehydrated LR-PRP), a blood-derived, anti-inflammatory protein solution containing multiple anti-inflammatory cytokines as well as growth factors, prepared from a small sample of a patient's blood, has been reported as an effective autologous treatment for osteoarthritis [15]. However, the differences in cell compositions and cytokine levels between APS and other PRPs have not been reported yet. Therefore, the present study was aimed to compare the quality of these two different types of PRPs, APS, and LP-PRP, with respect to cell composition and cytokine levels in HV. In addition, we compared the adverse effects of these two types of PRP injections in KOA patients by retrospectively reviewing the clinical records.

## 2 Materials and methods

The procedures in this study were approved by the Institutional Review Board of our institution. Informed consent was obtained from each donor and patient before drawing peripheral blood. Cell composition, growth factors, and cytokines contained in APS and LP-PRP were analyzed in ten healthy male volunteers (HV). In addition, the clinical records, including the cell composition in PRPs were retrospectively reviewed in 16 KOA patients (22 knees) who underwent both LP-PRP and APS therapy at our hospital. The protocol and ethics of this treatment were certified by a special committee for regenerative medicine based on a law regarding the safety of regenerative medicine in Japan. At our institution, though both LP-PRP and APS were approved for the treatment of KOA, LP-PRP is the first choice, and APS is used specifically used when the patient wants to use APS or does not respond to LP-PRP injection, or who wishes further improvement after LP-PRP injection. Some patients underwent PRP therapy to their both knees following the same protocol; additionally, in these cases, the adverse effects were observed on each knee. The levels of growth factors and cytokines in PRPs were not evaluated in these KOA patients as these analyses were not included in the approved PRP therapy protocol, and need a large amount of sample as well.

### 2.1 Blood collection

Peripheral blood from HV and KOA patients was collected by experts using a 21-gauge needle. For APS preparation, 55 mL of the blood was put into 60 mL syringes containing 5 mL of anticoagulant citrate dextrose solution-A (ACD-A), for LP-PRP preparation, 22 mL was added to an LP-PRP preparation kit (MyCells^®^; Kaylight Technologies, Ltd., Holon, Israel), and for whole blood (WB) cell composition analysis, 2 mL blood was added into an EDTA-coated tube (TERUMO, Tokyo, Japan).

### 2.2 APS and LP-PRP preparation

APS was prepared following a previous study [15] using the APS kit (Zimmer Biomet, US). The APS Kit is a sterile single-use unit containing two blood processing devices with a vial of ACD-A. Briefly, 55 mL of blood was put into 60 mL syringes containing 5 mL ACD-A. After ensuring thorough mixing, the contents of the 60 mL syringe were transferred to a disposable separation device (APS kit; Zimmer BIOMET) containing a tuned density buoy, according to the manufacturer's instructions (IFU 01-50-1456). After an initial centrifugation step at 1800 × *g* for 15 min at 21‒25 °C, platelet-poor plasma (PPP) was collected, and PRP was aspirated from the device. PRP was then transferred into the concentrator containing dried polyacrylamide beads for dehydration and further processed by centrifugation at 1125 × *g* for 2 min. The 3 mL output was collected as APS.

Peripheral blood (22 mL) that was collected in a MyCells^®^ PRP preparation kit was transferred into two sets of MyCells^®^ PRP harvesting kits (11 mL each). After centrifugation at 2000 × *g* for 7 min at 21‒25 °C, the supernatant was discarded, and the remaining 2 mL of plasma from each kit was collected as LP-PRP (4 mL in total) after pipetting out the buffy-coat layer. The PPP, LP-PRP, and APS harvested from HV were stored at −80 °C until further analyses.

### 2.3 Hematological analysis

Cell counts (platelet, leukocyte, and erythrocyte) and cell composition of leukocytes in WB, PPP, LP-PRP, and APS were measured using an automated hematology analyzer (Ac-T diff; Beckman Coulter, Brea, CA, USA).

### 2.4 Quantification of growth factors and cytokines

The concentrations of one growth factor (PDGF-BB) and four cytokines—interleukin 1 receptor antagonist (IL-1Ra), soluble TNF receptor type II (sTNF-RII), interleukin 1-β (IL-1β), and tumor necrosis factor-α (TNF-α)—in each PPP, LP-PRP, and APS sample were measured. All growth factor and cytokine quantification assays were performed using an enzyme-linked immunosorbent assay kit (PDGF-BB: Abcam System, Japan, ab181421; TNF-α: Abcam System, ab214025; sTNF-RII: Abcam System, ab184860; IL-1β: R&D Systems, US, DRT200; IL-1Ra: R&D Systems, DRA00B) without dilutions. All procedures were carried out according to the manufacturers' instructions.

### 2.5 Review of the clinical record

The clinical records of the KOA patients who underwent both LP-PRP and APS therapy at our hospital from May 2018 to March 2020 were reviewed, and adverse effects such as infection and arthralgia were evaluated.

### 2.6 Statistical analysis

All data are presented as the mean ± SD. In HV, comparisons of all pairs in each group were assessed using a nonparametric one-way analysis of variance followed by Tukey's *posthoc* test. In KOA, the comparison of all pairs in each group was assessed using paired *t*-test and Fisher's exact test. All *p*-values were two-sided, and *p*-values <0.05 were considered significant. Statistical analyses were performed with Graph Pad Prism version 8.0 (GraphPad Software, Inc., La Jolla, CA, USA).

## 3 Results

### 3.1 Characteristics of HV and KOA patients

The characteristics of the HV and KOA patients are summarized in Table 1. The mean age of the HV was 33.6 yrs, while that of the KOA patients was 70.5 yrs.

**Table 1:** Characteristics of healthy volunteers (HV) and knee osteoarthritis (KOA) patients.

Characteristics	HV	KOA
Age in yrs (range)	33.6 (31‒38)	70.5 (56‒82)
Sex	10 males	4 males (5 knees)
		12 females (17 knees)
BMI (Mean ± SD)	23.2 ± 1.6	24.6 ± 3.0
Erythrocyte (×10^4^/µl)	451.0 ± 81.9	463.4 ± 50.2
Leukocyte (×10^3^/µl)	4.3 ± 1.1	5.9 ± 1.0
Platelet (×10^4^/µl)	18.7 ± 3.1	25.7 ± 8.4

### 3.2 Platelet and leukocyte concentration

**Fig. 1:**
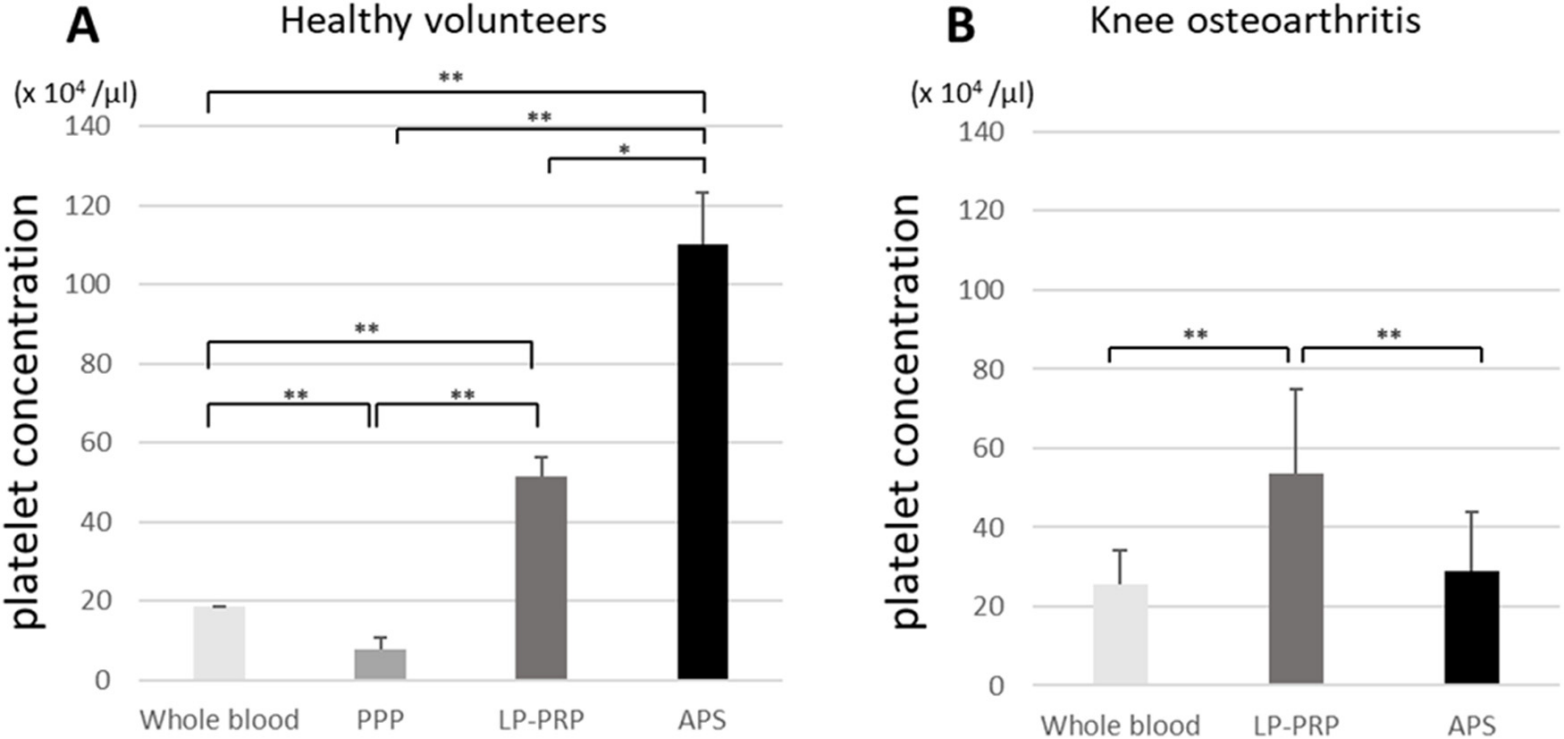
Platelet concentrations in healthy volunteers (HV) (A), and knee osteoarthritis (KOA) patients (B). Data are presented as the mean ± SD (**p < 0.01, *p < 0.05). PPP, platelet-poor plasma; LP-PRP, leukocyte-poor platelet-rich plasma; APS, autologous protein solution.

In HV, the platelet concentration in APS (110.1 ± 47.2 × 10^4^/µl) was significantly higher than that in PPP (7.6 ± 4.8 × 10^4^/µl; *p* < 0.01), WB (18.7 ± 3.1 × 10^4^/µl; *p* < 0.01), and LP-PRP (51.3 ± 13.0 × 10^4^/µl; *p* < 0.05), whereas, in KOA it was maximum in LP-PRP (53.4 ± 21.5 × 10^4^/µl) followed by APS and WB (29.0 ± 14.9 × 10^4^/µl, 25.7 ± 8.4 × 10^4^/µl; *p* < 0.01) (Fig. 1).

**Fig. 2:**
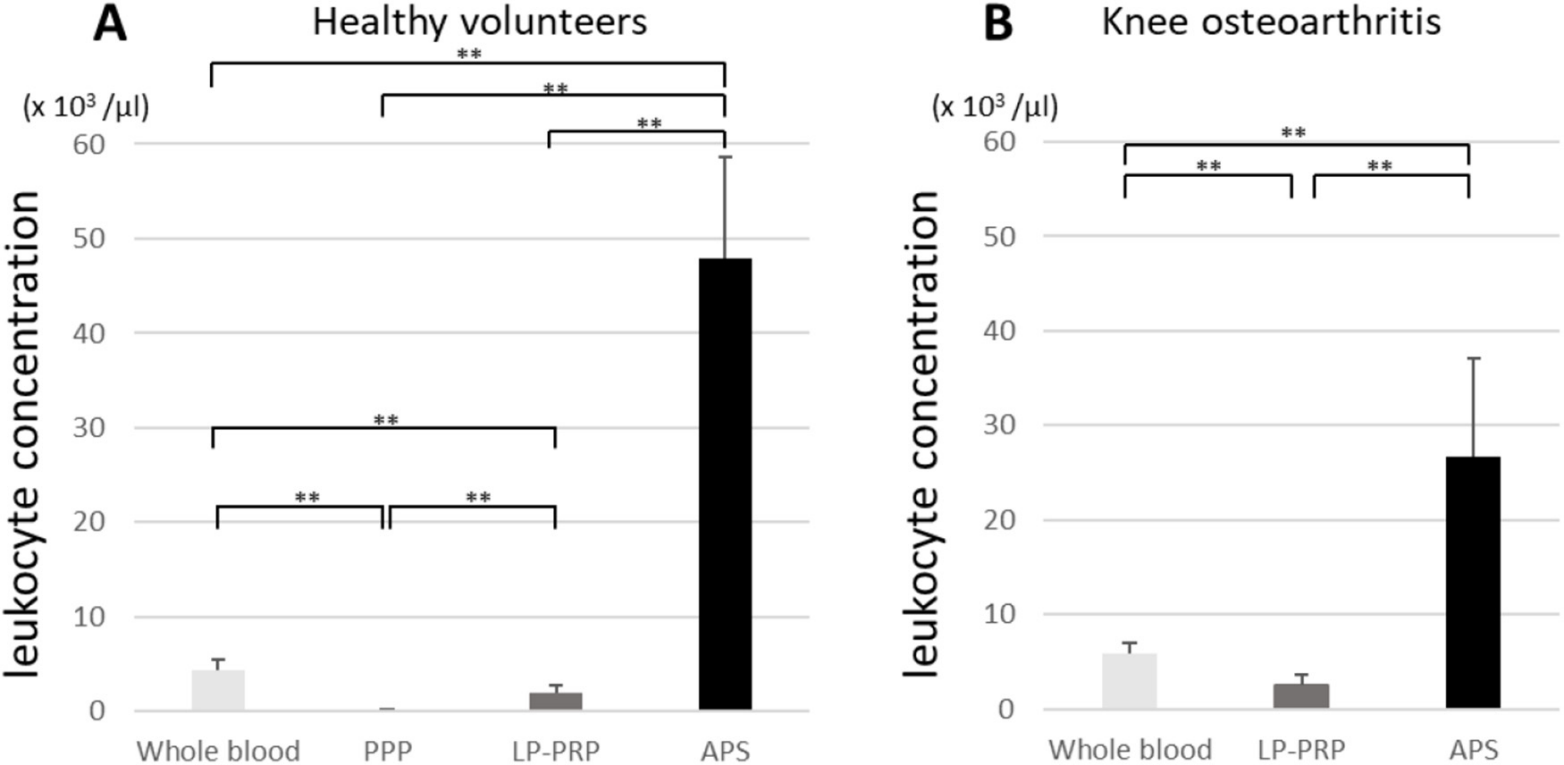
Leukocyte concentrations in healthy volunteers (HV) (A), and knee osteoarthritis (KOA) patients (B). Data are presented as the mean ± SD (**p < 0.01, *p < 0.05). PPP, platelet-poor plasma; LP-PRP, leukocyte-poor platelet-rich plasma; APS, autologous protein solution.

In both HV and KOA patients, the leukocyte concentration in APS was maximum (44.1 ± 7.5 × 10^3^/µl, and 26.6 ± 10.5 × 10^3^/µl, respectively) and differed significantly from that in PPP (0.1 ± 0.06 in HV; *p* < 0.01), WB (4.3 ± 1.1 × 10^3^/µl, 5.9 ± 1.0 × 10^3^/µl; *p* < 0.01), and LP-PRP (1.9 ± 0.8 × 10^3^/µl, 2.4 ± 1.1 × 10^3^/µl; *p* < 0.01) (Fig. 2).

Following the PAW classification system [11], LP-PRP was classified as P2-B type based on the estimated concentrations of platelets and leukocytes, while APS was classified as P3-A type and P2-A type in HV and KOA, respectively.

### 3.3 Leukocyte composition

In WB, LP-PRP, and APS the leukocytes were observed to comprise lymphocytes, monocytes, and neutrophils (Tab. 2). Among which, lymphocytes were predominant in LP-PRP in HV (89.7%) as well as KOA patients (84.8%), and neutrophils were predominant in APS (40.1%, 38.5%, respectively). The content of lymphocytes (47.9%, 52.1% in HV, and KOA, respectively) was mostly similar. In other words, the composition of leukocytes showed a similar trend between HV and KOA patients in WB, LP-PRP, and APS. According to these analyses, APS contained a high number of all types of leukocytes, whereas LP-PRP contained a low number of leukocytes with lymphocytes predominant (Tab. 2).

**Table 2:** Leukocyte concentration and composition in whole blood (WB), LP-PRP, and APS.

PRPs and Blood components	Healthy volunteers (HV)				Knee osteoarthritis (KOA)			
	Leukocyte concentration (×10^3^/ml)	Cell counts (×10^3^/ml) and composition (%)			Leukocyte concentration (×10^3^/ml)	Cell counts (×10^3^ /ml) and composition (%)		
		Lymphocyte	Monocyte	Neutrophil		Lymphocyte	Monocyte	Neutrophil
Whole blood	4.3 ± 1.1	1.4 ± 0.3 (33.3 ± 8.4)	0.2 ± 0.09 (5.9 ± 2.0)	2.7 ± 1.1 (60.7 ± 9.0)	5.9 ± 1.0	1.7 ± 0.3 (29.2 ± 4.7)	0.3 ± 0.1 (6.5 ± 2.5)	3.8 ± 0.8 (64.2 ± 4.1)
LP-PRP	1.9 ± 0.8	1.7 ± 0.7 (89.7 ± 3.0)	0.1 ± 0.06 (5.4 ± 2.8)	0.08 ± 0.09 (3.7 ± 2.9)	2.4 ± 1.1	2.3 ± 0.8 (84.8 ± 5.4)	0.2 ± 0.1 (7.5 ± 6.5)	0.2 ± 0.2 (7.5 ± 4.7)
APS	44.1 ± 7.5	20.6 ± 3.2 (47.9 ± 10.7)	5.0 ± 3.2 (11.8 ± 8.0)	18.4 ± 9.4 (40.1 ± 14.8)	26.6 ± 10.5	13.1 ± 3.8 (52.1 ± 15.3)	2.4 ± 1.1 (9.2 ± 2.7)	11.1 ± 7.0 (38.5 ± 16.1)

Data are presented as the mean ± SD. LP-PRP, leukocyte-poor platelet-rich plasma; APS, autologous protein solution.

### 3.4 Quantification of growth factors and cytokines in PPP, LP-PRP, and APS

**Fig. 3:**
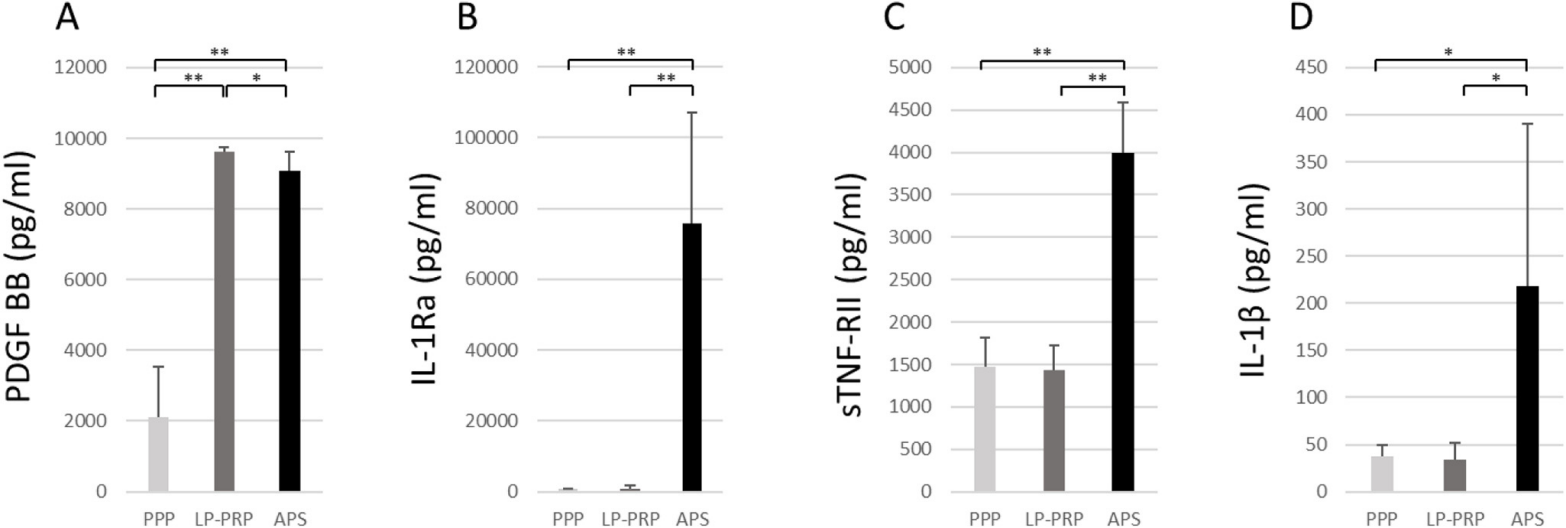
Concentrations of platelet-derived growth factor-BB (PDGF-BB) (A), and cytokines, including IL-1 receptor antagonist (IL-1 Ra) (B), soluble TNF receptor type II (sTNF-RII) (C), and IL-1β (D). Data are presented as the mean ± SD (**p < 0.01, *p < 0.05).

**Fig. 4:**
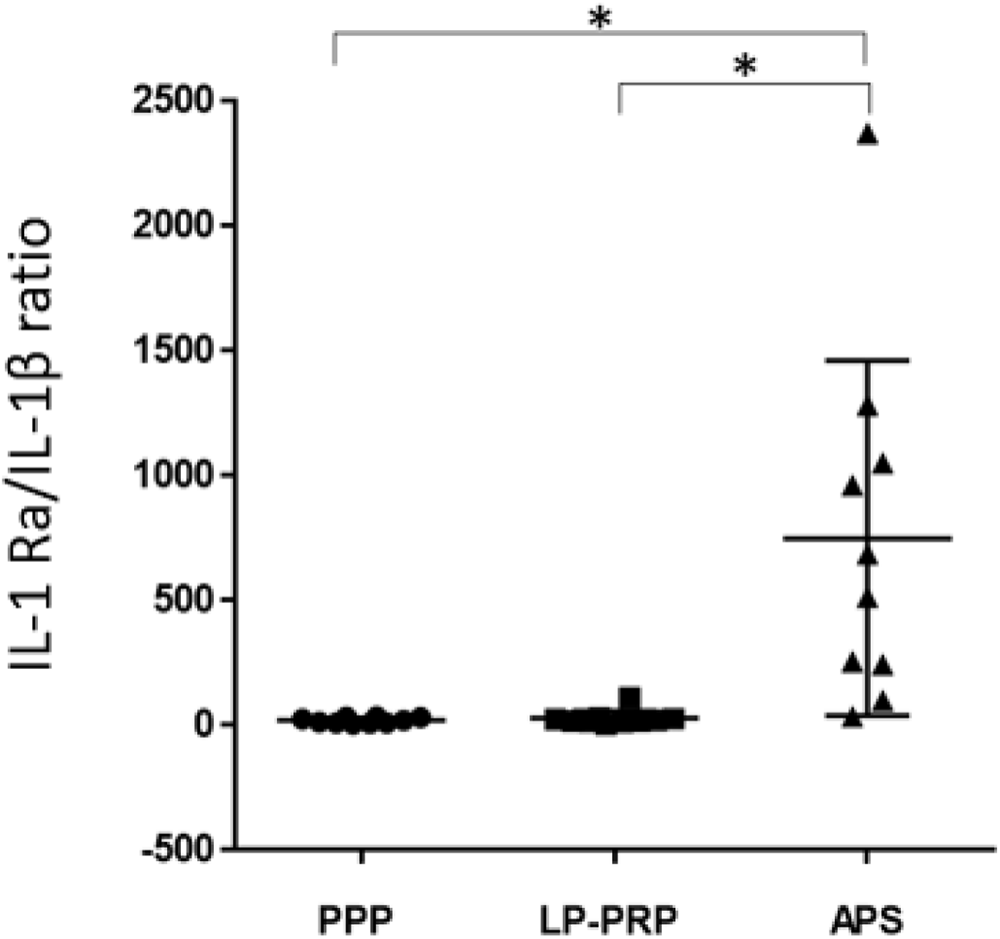
The ratio of anti-inflammatory/inflammatory cytokine. Data are presented as mean ± SD, *p < 0.01.

The quantification of growth factors and cytokines in PPP, LR-PRP, and APS is summarized in Figure 3. The concentration of the growth factor, PDGF-BB, was significantly higher in APS and LP-PRP than in PPP (Fig. 3A). Interestingly, although platelet concentration in APS was approximately two-fold higher than that in LP-PRP, the PDGF levels in APS were significantly lower than those in LP-PRP (*p* = 0.02). The concentrations of anti-inflammatory cytokines, IL-1Ra (Fig. 3B), and sTNF-RII (Fig. 3C) were found to be significantly higher in APS than in LP-PRP (*p* < 0.01). The concentrations of the pro-inflammatory cytokines such as IL-1β were significantly higher in APS (*p* = 0.02) than those in PPP and LR-PRP (Fig. 3D). However, TNFα was undetectable in most of the samples (data not shown). Furthermore, the anti-inflammatory cytokine/inflammatory cytokine ratio represented by the ratio of IL-1Ra/IL-1β was significantly higher in APS (750.8 ± 710.5) than in PPP (20.3 ± 11.5) and LP-PRP (28.8 ± 30.4) (*p* < 0.01) (Fig. 4).

### 3.5 Review of the clinical records of patients who received both APS and LP-PRP injections

Sixteen patients (22 knees) underwent both APS and LP-PRP therapy in our hospital. The review of clinical records of these patients revealed that no major complications, such as infection, were observed during the treatment and follow-up period. However, some minor complications, such as arthralgia, were observed following injections with APS and LP-PRP. The incidence of arthralgia was significantly higher, and its duration was longer after the APS injection than that with the LP-PRP injection (Tab. 3). However, the clinical outcomes of APS treatment have not been assessed yet, as the mean follow-up period was less than 6-months.

**Table 3:** Adverse events observed after LP-PRP and APS injections in KOA patients.

Adverse events	LP-PRP	APS	Statistical analysis
Infection	0	0	NA
Arthralgia	3 (13.6%)	11 (50%)	Fisher's exact test *p* = 0.02
Duration of arthralgia in days (range)	1.33 (1‒2)	3.81 (1‒7)	Paired-*t* test *p* < 0.01

NA, not applicable.

## 4 Discussion

Here, using a controlled laboratory study and a retrospective observational study, we have demonstrated the difference between two different types of PRPs, APS and LP-PRP. These two PRPs vary with respect to their cell compositions and cytokine levels. In addition, we evaluated the PRPs prepared through different methods and showed that the methods used for the preparation of APS and LP-PRP determine the quality and efficacy of PRP therapy.

Several studies investigating the cell compositions, cytokines, and growth factors in PRPs prepared using different types of PRP preparation systems have reported that the efficacy could differ depending on the preparation methods [11,16,17]. Here, we showed that the cell compositions of LP-PRP and APS were quite different. APS contained high concentrations of leukocytes, high levels of IL-1β, IL-1Ra, and sTNF-RII, and had a high IL-1Ra/IL-1β ratio. The results were supported by O'Shaughnessey et al. [15], who reported that APS contains high concentrations of IL-1 Ra and sTNF-RI, which block IL-1β and TNFα. It has also been reported that IL-1Ra inhibits MMP-13 production in chondrocytes stimulated with IL-1β and TNFα in a dose-dependent manner [18]. Taken together, APS was categorized as a novel LR-PRP with high levels of anti-inflammatory cytokines.

In this study, the platelet concentration in APS from KOA patients was lower than that from HV, which could be attributed to the difference between the ages of the patients. However, the levels of anti-inflammatory cytokines and growth factors have been reported to be similar in APS from KOA patients and HV [19]. These observations may indicate that platelets were activated during the APS preparation process, and growth factors and cytokines had already been released from platelets. Therefore, though the platelet count was low in APS from elderly KOA patients, the levels of growth factors were similar in both HV and KOA patients. However, there is a possibility that the growth factors in PRPs could be affected by the age of the patients. Therefore, further study with more samples is required to investigate the growth factors and cytokines in PRPs.

In this study, IL-1β levels in APS were higher than those in LP-PRP. Therefore, we speculated that the administration of APS into the joint would provoke the inflammatory response, such as reactive synovitis. To the best of our knowledge, here, for the first time, we have compared the adverse events in response to APS and LP-PRP injection in the same individual and showed that there were a higher incidence and longer duration of arthralgia after APS injection as compared to the LP-PRP injection (Tab. 3). However, Kon et al. have reported no significant difference in the adverse events between the APS and the controlled saline injection group [20]. This disparity could be due to the difference between the levels of anti- and pro-inflammatory cytokines in APS from different individuals. Moreover, the response of patients could differ depending on their characteristics, such as sex, age, comorbid conditions, and race. The use of APS for KOA treatment is increasing in Japan, its efficacy and adverse effect could be revealed using a large sample size in the Japanese population.

This study had several limitations. First, the sample size was small, which might have affected the results. However, based on the statistical power analysis (alpha = 0.05 and power = 0.8), the minimum sample size to determine the differences were the following: five subjects for the levels of IL1-Ra, three subjects for sTNF-RII, and thirteen subjects for IL-1β. Therefore, the statistical power was sufficient to at least confirm the results of IL-1Ra and sTNF-RII concentrations. Second, growth factors and cytokines were measured only in HV because the APS from KOA patients had been collected for clinical use, and we retrospectively investigated the data of cell compositions in APS from clinical records. Third, growth factors and cytokines in LR-PRP were not measured. As the white blood cell counts in LR-PRP were similar to those in APS, measurement of cytokine levels in LR-PRP could provide information on whether an additional step of filtration through polyacrylamide beads is important.

## 5 Conclusions

This study showed that the PRP preparing devices influenced the quality of PRP, which could affect their clinical treatment outcomes. APS contains similar levels of growth factors compared to conventional LP-PRP but contained very high concentrations of anti-inflammatory cytokines. Therefore, APS could serve as a novel treatment option for KOA, and especially for patients with excessive synovial fluid and non-responders to conventional PRP therapy. Clinicians should recognize these differences among PRP preparations and choose an appropriate system depending on the pathophysiology of the patients. However, further *in vitro* and *in vivo* studies are needed to elucidate the differences between the biological effects of APS and other PRPs.

## Conflict of Interest

The authors declare no conflict of interest.
